# ASSOCIATION OF IMMUNE CELLS AND INFLAMMATORY BIOMARKERS WITH DEPRESSIVE SYMPTOMS IN THE FRAMINGHAM HEART STUDY

**DOI:** 10.1093/geroni/igae098.3803

**Published:** 2024-12-31

**Authors:** Sandhya Iyer, Yumeng Cao, Kathryn Lunetta, Ryan Liebegott, Margaret Doyle, Jiachen Chen, Ahmed Ragab, Joanne Murabito

**Affiliations:** Boston University School of Medicine, Boston, Massachusetts, United States; Boston University, Boston, Massachusetts, United States; Boston University, Boston, Massachusetts, United States; Boston University School of Public Health, Boston, Massachusetts, United States; University of Vermont Larner College of Medicine, Colchester, Vermont, United States; Boston University School of Public Health, Boston, Massachusetts, United States; Boston University, Boston, Massachusetts, United States; Chobanian and Avedisian School of Medicine, Boston University, Boston, Massachusetts, United States

## Abstract

Depression is a common mental health disorder with substantial impact on older adults. Immune dysfunction has been studied as a mechanism for depressive symptoms, providing the opportunity for new targeted treatment approaches. This study investigated the associations between immune cell phenotypes, determined by flow cytometry, and peripheral inflammatory biomarkers, measured using the OLINK Inflammation panel, with Center for Epidemiologic Studies Depression Scale (CES-D) scores, indicative of depressive symptoms, in the Framingham Heart Study Offspring Cohort. Participants (n=996) had a mean age 62, were 52% female, and had a mean CES-D score 5 (SD=7) with 8% having a CES-D score >/=16. A linear mixed-effects model was used (generalized estimating equation model for the dichotomous CES-D) with the proteins and immune cells as predictors and CES-D scores as the outcome, adjusting for age, sex, BMI, smoking status, CVD, depression medication use, NSAID use, and cytomegalovirus (CMV) status (immune cells only). We used Bonferroni adjusted p< 0.005 to declare significance. Effect estimates (beta) are reported in standard deviation units. Our results suggested a positive association between FGF-19 and CES-D score (beta=0.11, p=0.0004), as well as a suggestive negative trend between the CD8+IL17+:CD8+CD25+FoxP3+ ratio (Tc17/CD8+Tregs) and CES-D score (beta=-0.1, p=0.03). In addition, we observed a positive suggestive trend between IL-17C and depressive symptoms (CES-D >/= 16) (beta=0.35 p=0.009), that strengthened with higher levels of symptoms (p< 0.005). This study helps to expand our understanding of the role of inflammation in the pathophysiology of depression.

